# Utilization of Different Omic Approaches to Unravel Stress Response Mechanisms in the Parasite *Entamoeba histolytica*

**DOI:** 10.3389/fcimb.2018.00019

**Published:** 2018-02-08

**Authors:** Shruti Nagaraja, Serge Ankri

**Affiliations:** Department of Molecular Microbiology, Ruth and Bruce Rappaport Faculty of Medicine, Technion, Haifa, Israel

**Keywords:** *Entamoeba histolytica*, omics, oxidative stress, nitrosative stress, iron starvation, glucose starvation, microbiota, virulence

## Abstract

During its life cycle, the unicellular parasite *Entamoeba histolytica* is challenged by a wide variety of environmental stresses, such as fluctuation in glucose concentration, changes in gut microbiota composition, and the release of oxidative and nitrosative species from neutrophils and macrophages. The best mode of survival for this parasite is to continuously adapt itself to the dynamic environment of the host. Our ability to study the stress-induced responses and adaptive mechanisms of this parasite has been transformed through the development of genomics, proteomics or metabolomics (omics sciences). These studies provide insights into different facets of the parasite's behavior in the host. However, there is a dire need for multi-omics data integration to better understand its pathogenic nature, ultimately paving the way to identify new chemotherapeutic targets against amebiasis. This review provides an integration of the most relevant omics information on the mechanisms that are used by *E. histolytica* to resist environmental stresses.

## Introduction

**Figure 1:**
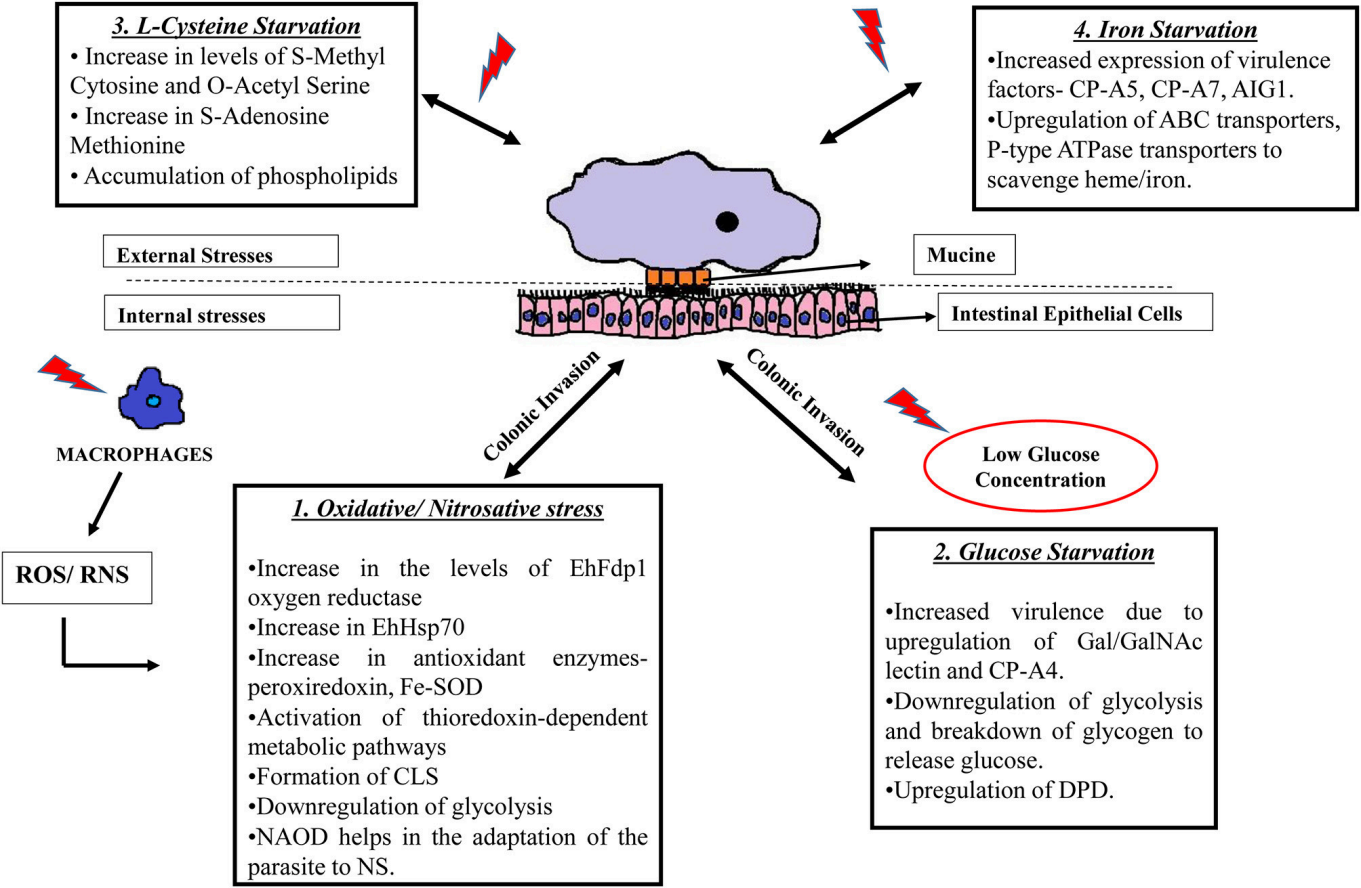
Strategies used by *E. histolytica* when challenged with different stresses. *E. histolytica* faces threat in a number of ways: 1. Following colonic invasion, trophozoites penetrate the intestinal epithelial layer of the host where they are challenged by immune cell during invasion. Exposure to ROS (Rastew et al., 2012) and RNS (Kolios et al., 2004) released by macrophages and other immune cells eventually triggers the parasite defense mechanisms as shown in the box. 2. The amoeba is also threatened by low glucose concentration in the colon and it survives this condition by inhibiting glycolysis and by degrading stored glycogen and converting it to free glucose. Moreover, low glucose concentration triggers the parasite to become more virulent by upregulating Gal/GalNAc lectins (Baumel-Alterzon et al., 2013). 3. Changes occurring during the absence of L-cysteine, which is an important thiol required for antioxidant activity in the parasite. During cysteine starvation, there is an increase of phospholipids and other metabolites such as S-methyl cytosine, S-adenosine methionine etc. (Jeelani et al., 2014). 4. The absence of iron also increases virulence of the parasite by increasing the expression of CP-A5, CP-A7, and leads to the upregulation of transport proteins to scavenge iron from other external sources (Hernandez-Cuevas et al., 2014). SOD, superoxide dismutase; CLS, cyst like structure; NAOD, N-acetyl ornithine deactylase; Gal/GalNAc, Galactose/N-acetylgalactosamine binding lectin; CP, cysteine proteinases; DPD, dihydropyramidine dehydrogenase; AIG, Androgen Inducible Gene; ABC transporters; ATP, binding cassette transporters.

**Figure 2:**
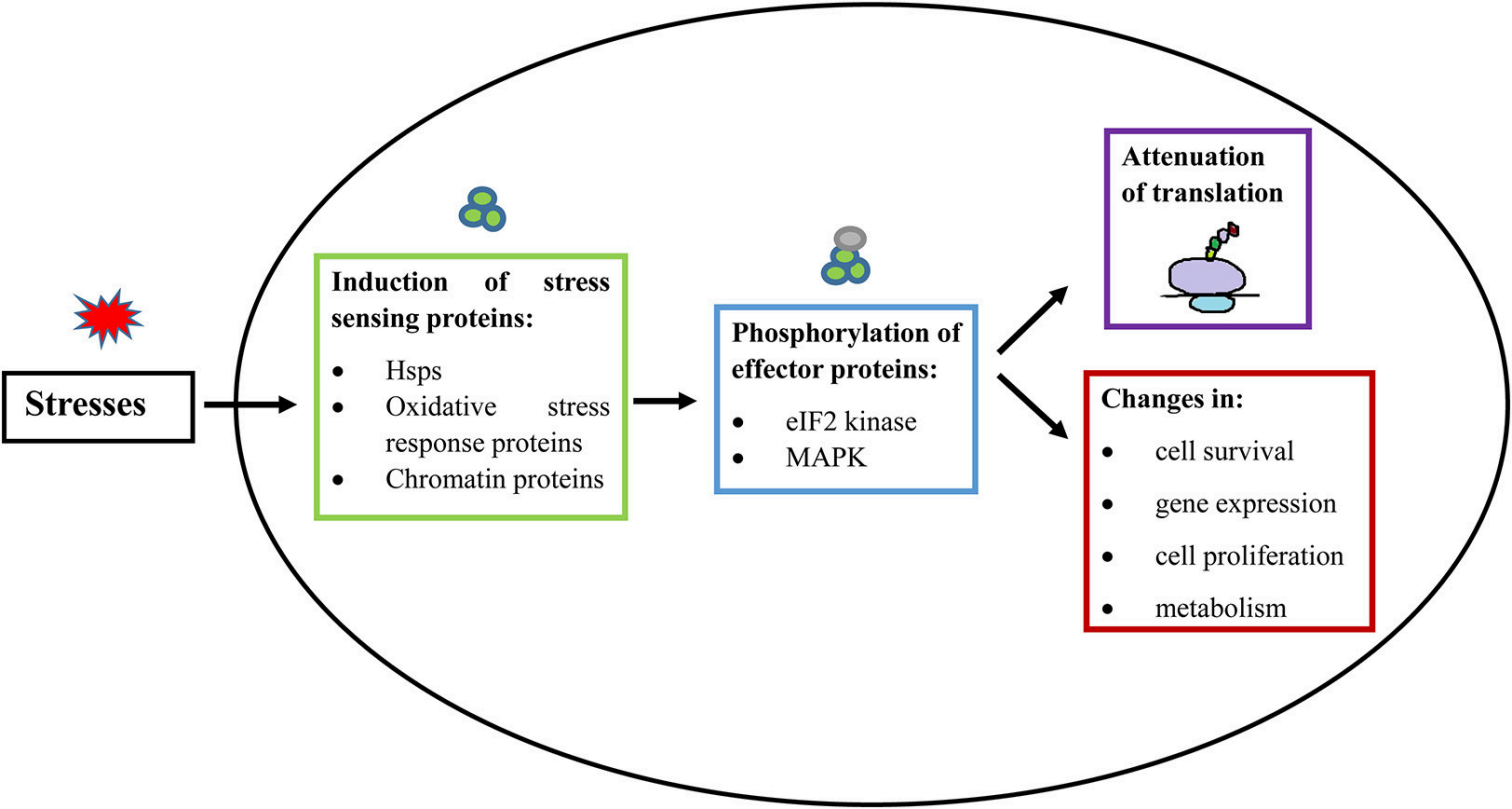
General principle of stress response in eukaryotic cells and *E. histolytica*. When a cell is exposed to stress, there is an activation of a stress-responsive protein (De Nadal et al., 2011; Walter and Ron, 2011; Smith and Workman, 2012), which induces signal transduction mediated by protein phosphorylation (Darling and Cook, 2014; Sharma et al., 2016). The stress signal is then transferred to downstream effector proteins. These stress responses generally lead to inhibition of cap-dependent translation initiation and consequent suppression of general protein synthesis. Stress responses also lead to additional changes, involving modulation of gene expression, cell proliferation, cell survival, and changes in metabolism (Lopez-Maury et al., 2008). Hsps, Heat shock proteins; eIF2, Eukaryotic Intitation Factor 2; MAPK, Mitogen Activated Protein Kinases.

Amebiasis, caused by the eukaryotic parasite *Entamoeba histolytica*, is an enormous global medical problem that still exists due to poor sanitary and unhygienic conditions. According to the World Health Organization, 50 million people in India, Southeast Asia, Africa, and Latin America suffer from amebic dysentery and liver abscesses, and amebiasis causes the death of at least 100,000 individuals each year. In 90% of the infected patients, *E. histolytica* trophozoites normally inhabit the colon and spend their time in the host as a non-pathogenic commensal. However, the reasons why these trophozoites become virulent and invasive are unknown. Anti-amoebic drugs are the preferred choice due to the unavailability of vaccines. Based on their site of action, two categories of anti-amoebic drugs are used, namely, luminal amebicides (diloxanide furoate, and Iodoquinol) (Marie and Petri, 2013) and tissue amebicides (metronidazole) (Salles et al., 2007; Tazreiter et al., 2008; Marie and Petri, 2013) and potential resistance of the parasite to metronidazole is a real concern. Moreover, metronidazole is not effective in eliminating cysts inside the lumen and thus a combination of luminal and tissue amebicides is generally recommended (Marie and Petri, 2013). Recently, auranofin has been identified as a potent drug that targets redox enzymes in the parasite, eventually leading to oxidative stress in the parasite and it has found to be more effective than metronidazole (Debnath et al., 2012). Nevertheless, it is still a need of the hour to identify more potential drug targets to treat amebiasis. *E. histolytica* is challenged in the host environment due to fluctuations in partial pressure of oxygen, changes in glucose concentration and changes in the composition of the microbiota. The activation of innate immune responses against the parasite leads to the production of reactive oxygen species (ROS), nitric oxide (NO) by macrophages, complement activation and phagocytosis, and heat shock responses (Mortimer and Chadee, 2010; Moonah et al., 2013; Nakada-Tsukui and Nozaki, 2016; Olivos-Garcia et al., 2016). The parasite must be capable of adapting to the demand of surrounding environment in order to survive. This adaptive response of the parasite provides a shield against the host response as well as aids in their survival (Figure 1). In eukaryotic cells, the general stress response mechanism is a tightly orchestrated process. The first step involves the role of a stress-sensor proteins (heat shock proteins, nutrient sensing proteins, antioxidant proteins and also chromatin—proteins) to relay the message to the cells to adapt to stress (De Nadal et al., 2011; Walter and Ron, 2011; Santi-Rocca et al., 2012; Smith and Workman, 2012; Shahi et al., 2016a; Figure 2). The transfer of this stress signal to downstream proteins leads to a signal transduction cascade. This cascade begins with the phosphorylation of effector proteins [eIF2 kinases and Mitogen Activating Protein Kinases (MAPK)], and these proteins are known to play a role during stress. This eventually helps the cells to adapt to the stress by either attenuating translation (by phosphorylation of serine residue of the α subunit of eIF2 leading to its inactivation) (Jiang and Wek, 2005; Hendrick et al., 2016; Sharma et al., 2016) or through the modulation of gene expression and metabolism (Vonlaufen et al., 2008; Darling and Cook, 2014). While conventional molecular techniques provided an outline, the gradual development and utilization of “omics” technologies and bioinformatics to study *E. histolytica* open new avenues to understand the complexity of its behavior under different conditions. For example, the field of DNA microarrays and proteomics have revolutionized our manner to assess the virulence of the parasite and its ability to cope with various stresses (Gilchrist et al., 2006, 2010; López-Camarillo et al., 2009), study the expression of different genes in the parasite exposed to UV radiation (Gilchrist et al., 2006; Weber et al., 2009), and assess the effects of metronidazole as a chemotherapeutic agent (Tazreiter et al., 2008). Moreover, there are other studies investigating the proteome of cell surface proteins and the excretory-secretory protein system of the parasite that may help in understanding its pathogenicity (Biller et al., 2014; Ujang et al., 2016). With the help of different (transcriptomic, genomic, and metabolomics) omics analysis, it has now become possible to study the responses of the parasite to various stresses during host invasion and these studies can provide several critical pieces of evidence as to how the parasite manages to survive inside the host (Table 1). Thus, it is essential to review all the data in order to characterize the mechanisms essential for stress response and identify potential drug targets against this parasite. This article presents an overview of recent advances that have been made by using various omics approaches to investigate stress response in *E. histolytica*, with focus on oxidative stress (OS) and nitrosative stress (NS).

**Table 1:** A list of the different omics approaches used to analyze stress responses in *E. histolytica*.

**References**	**Omics approach used**	**Type of stress**	**Summary of the study**
Vicente et al., 2009	Transcriptomics	Oxidative stress	Upregulation of Hsps, MGL-1, DNA repair proteins Increase in the expression of signaling and regulatory proteins Upregulation of Fe-S flavoproteins
Jeelani et al., 2014	Metabolomics	Oxidative stress	Increased glycerol-3-phosphate levels Upregulation of components of chitin biosynthesis pathway Inactivation of key glycolytic enzymes
Davis et al., 2006	Comparative proteomics	Oxidative stress	Rahman strain deficient in peroxiredoxin and superoxide dismutase compared to the HM1:IMSS strain
Vicente et al., 2009	Transcriptomics	Nitrosative stress	Increase in expression of DNA damage repair proteins Upregulation of Hsps, MGL-1 Upregulation of phospholipid-Transporting-P-type ATPase Upregulation of signaling and regulatory proteins
Hertz et al., 2014a,b	Proteomics	Nitrosative stress	S-nitrosylation of cysteine residue of heavy subunit of Gal/GalNAc Lectin
Santi-Rocca et al., 2012	Proteomics	Nitrosative stress	Inactivation of glycolytic enzymes Low levels of ATP Fragmentation of ER
Tovy et al., 2011	Proteomics	Glucose Starvation	Upregulation of Gal/GalNAc lectin Upregulation of DPD
Baumel-Alterzon et al., 2013	Transcriptomics	Glucose starvation	Upregulation of DPD, MGL-1 Upregulation of virulence factors such as Gal/GalNAc lectins Downregulation of glycolytic genes
Husain et al., 2011	Transcriptomics	Cysteine Starvation	Upregulation of Fe-S family of proteins Accumulation of phosphatidylisopropanolamine
Husain et al., 2010	Metabolomics	Cysteine Starvation	Accumulation of S-methyl cysteine Upregulation of metabolites
Hernandez-Cuevas et al., 2014	Transcriptomics	Iron Starvation	Upregulation of cysteine proteinases, ribosomal proteins, elongation factors Upregulation of AIG1 Upregulation of NADH-dependent oxidoreductases, transport proteins
Weber et al., 2006	Transcriptomics	Heat Shock	Upregulation of Hsp70, CP-4, CP-6 Differential allelic expression of Gal/GalNAc lectin
Weber et al., 2009	Transcriptomics	UV irradiation	Upregulation of Fe-S proteins and DNA repair protein

*MGL-1, methyl-gamma-lyase-1; ATP, Adenosine-triphosphate; ER, Endoplasmic reticulum; DPD, dihydropyramidine dehydrogenase; Gal/GalNAc lectin, Galactose N-acetylgalactosamine lectin; AIG-1, Androgen Inducible gene; Fe-S, Iron sulfur cluster proteins*.

## Oxidative stress—what triggers it?

The key players that are involved in OS are a variety of ROS. They are capable of damaging essential biomolecules in the cell such as DNA, proteins, lipids and they lead to the fragmentation of the endoplasmic reticulum (ER) due to the accumulation of misfolded proteins (Imlay, 2003; Apel and Hirt, 2004; Pineda and Perdomo, 2017). In the large intestine, the invading *E. histolytica* trophozoites encounter OS. The sources of these stresses are fluctuations in oxygen tension in the intestinal lumen and the generation of ROS by cells of the immune system. Hydrogen peroxide (H_2_O_2_) is capable of damaging the proteins by its interaction with thiol groups, which are present in the cysteine side chains as well as with metal cofactors. Once formed, ROS leads to the oxidative damage of proteins thereby affecting their structure and functional properties (Shacter, 2000; Wu et al., 2006; Aiken et al., 2011). OS resistance contributes to the pathogenic potential of *E. histolytica* (Rastew et al., 2012).

### How did omics help us understand oxidative stress response in *E. histolytica*?

Analysis of the parasite's transcriptome in response to OS showed that this parasite copes with this stress by a complex modulation of a broad set of genes encoding proteins that are mainly involved in protein folding [Heat shock proteins (Hsps)], amino acid catabolism (MGL-1), signaling/regulatory pathways, and also in pathways involved in repair during DNA damage and metabolism (Vicente et al., 2009). The authors reported an upregulation of Rad3 helicase, Rad50, and DNA excision base repair proteins. These proteins along with Rad52, are also involved in DNA damage response in the parasite during UV irradiation (Weber et al., 2009) Table 1. Two genes coding for deoxyuridine triphosphate nucletotidehydrolase (dUTPase) were upregulated in the parasite exposed to OS. These genes are considered essential for the stability of DNA and have been proposed as a potential drug target against parasites (Nguyen et al., 2005). A 4-fold increase in the homolog of polynucleotide kinase-3 phosphatase along with a 2-fold increase in MutS DNA repair proteins was also reported. These proteins are known for their role in repairing DNA breaks during formed during OS (Chang et al., 2002; Blondal et al., 2005). These proteins were also found to be upregulated when the parasite was exposed to NS.

A functional study of genes responsive to OS revealed the role of the stress-induced adhesion factor, the phospholipid-transporting P-type ATPase and the EhFdp1 oxygen reductase in the resistance of the parasite to OS (Rastew et al., 2012).

The role of Hsps in the resistance of the parasite to stresses has been widely credited as they facilitate the stabilization/sequestration of damaged or misfolded proteins (Kaul and Thippeswamy, 2011). Hsps are very well conserved in all organisms and they function as molecular chaperons during any stressful event (Perez-Morales and Espinoza, 2015). Hsp70 is known to aid in the refolding of denatured and misfolded proteins, and translocation of secretory proteins (Voisine et al., 1999). *E. histolytica* Hsp70 (EhHsp70) is essential for the resistance of the parasite to OS, the formation of liver abscess, and its levels are also upregulated during heat shock response in the parasite (Akbar et al., 2004; Weber et al., 2006; Santos et al., 2015) Table 1. EhHsp70 expression is also upregulated when the *E. histolytica* Dnmt2 homolog (Ehmeth) is overexpressed. Ehmeth catalyzes the methylation of C_38_ present in the anticodon loop of tRNA^Asp^ (Tovy et al., 2010). Ehmeth-overexpressing trophozoites exhibit significantly greater survivability to H_2_O_2_ exposure, which emphasizes the role of EhHsp70 in OS resistance suggesting that EhHsp70 expression is under epigenetic control (Fisher et al., 2006).

It has been recently reported that the gene expression under OS influence is regulated by a transcription factor that binds to a specific motif (AAACCTCAATGAAGA) in the promoters of the genes receptive to H_2_O_2_ (Pearson et al., 2013). It is interesting to note that there is an association between the expression of *E. histolytica* OS responsive genes and the parasite's virulence (Rastew et al., 2012). Antioxidant enzymes such as glutathione peroxidase, glutathione, and catalase provide a shield for an organism to defend the harsh conditions of OS. *E. histolytica* lacks the presence of these antioxidative enzymes (Tekwani and Mehlotra, 1999) and it relies mostly on two proteins for its defense against OS. These are the 29-kDa peroxiredoxin (Sen et al., 2007) and the iron peroxide dismutase (Fe-SOD) (Bruchhaus and Tannich, 1994). *E. histolytica* also relies on exclusive variants of antioxidants that have a low molecular mass like cysteine (Krauth-Siegel and Leroux, 2012). In contrast to *E. histolytica*, which utilizes superoxide dismutases (SOD), *Giardia intestinalis*—another common intestinal parasite, has also developed defense mechanisms to cope with OS. This parasite utilizes superoxide reductases (SORs) for the elimination of superoxide anions by reducing H_2_O_2_ (Testa et al., 2011). Moreover, this parasite contains high levels of NADH-dependent oxidase for detoxification of oxygen (Tekwani and Mehlotra, 1999). Pyruvate has also been identified as another alternate potential antioxidant protein that detoxifies H_2_O_2_ inside *Giardia spp* (Biagini et al., 2001).

An interesting study using capillary electrophoresis–mass spectrometry was performed to determine the effects of OS on the metabolism of the parasite (Husain et al., 2012). OS inactivates the glycolytic pathway and increases the production of glycerol along with changes in nucleotide metabolism, and activation of the chitin biosynthetic pathway. These data suggest that the glycerol synthesis pathway defends the parasite against OS and that oxidized proteins may be crucial constituents of the parasite's machinery to cope with OS. *E. histolytica* accounts on the thiol-dependent redox metabolism to resist OS (recently reviewed in Jeelani and Nozaki, 2016). The thioredoxin-dependent system has been extensively studied in *E. histolytica* and it consists of Fe-superoxide dismutase, rubrerythrin, peroxiredoxin, flavodiiron proteins, and amino acids including L-cysteine, and thioprolines (thiazolidine-4-carboxylic acids), and *S*-methyl-l-cysteine respectively (Jeelani and Nozaki, 2016). Some of the thiol-dependent redox metabolism proteins like the iron-containing superoxide dismutase have been found to be oxidized in trophozoites exposed to H_2_O_2_ (Shahi et al., 2016a). It was also reported that the presence of superoxide radical anions, which cause OS, lead to the expression of superoxide dismutase and these enzymes are known to contain iron (Bruchhaus and Tannich, 1994). These enzymes further interact with the metabolites of the drug metronidazole and form covalent adducts (Leitsch et al., 2007). The effect of oxidation on the activity of the amebic iron-containing superoxide dismutase has still not been determined yet. Proteomics has helped us to strengthen our understanding of the parasite's response to OS. Comparative proteomics of the virulent strain—HM1:IMSS and the avirulent strain—Rahman showed that Rahman is deficient in two proteins with antioxidative properties (peroxiredoxin and superoxide dismutase). Overexpression of peroxiredoxin in Rahman restores its resistance to OS and its ability to cause colitis in human colonic xenografts (Davis et al., 2006). Proteomics has also been extensively used to study the composition of cysts (Ali et al., 2012) and cyst-like structures (CLS) (Luna-Nacar et al., 2016). CLS is formed in response to OS in the parasite, and they may be part of the parasite's mechanisms to stand OS. CLS share features found in cysts like the presence of a chitin-like resistant coat on their surface and features of trophozoites exposed to OS like the expression of stress response proteins and redox homeostasis (Rastew et al., 2012) and a downregulation of glycolysis and metabolism-related proteins (Jeelani and Nozaki, 2016). Surprisingly, there is no proteomics data available in the literature about the parasite's response to OS and we are currently working to fill this knowledge gap. However, redox proteomics has been recently performed by using resin-assisted capture of oxidized proteins (OX-RAC) (Shahi et al., 2016a). OX-RAC does not provide information about the differential level of proteins before, and after exposure to OS, however, it somewhat identifies oxidized cysteines in proteins following the exposure of the parasite exposed to OS (Kohr et al., 2011). Some of the oxidized proteins identified by OX-RAC like superoxide dismutase have been associated with antioxidant activity. However, a very weak overlapping was found between genes that have their expression changed during OS (Vicente et al., 2009) and oxidized proteins identified by OX-RAC (Shahi et al., 2016a).

Taken together, these findings emphasize the importance of performing multi-omics approaches to fully understand the response of the parasite exposed to stress.

## Nitrosative stress—how does the parasite cope with it?

A second challenge encountered by *E. histolytica* presents itself in the form of reactive nitrogen species (RNS) in the large intestine. Here the parasite is exposed to NO at nanomolar concentrations by the cells of intestinal epithelium (Kolios et al., 2004). During acute inflammation, the activation of specific immune cells of the host's immune system comprising of natural killer cells, macrophages, and neutrophils releases NO in micromolar amount (Thomas et al., 2008; Begum et al., 2015). S-nitrosylation is a post-translational modification that occurs by covalent attachment of NO group to the thiol side chain of cysteine residues in proteins (Hess et al., 2005). NO-mediated cytotoxicity is partly due to the formation of S-nitrosylated proteins which results in aberrant protein function. For example, the inactivation of key glycolytic enzymes in the parasite exposed to NO leads to the fragmentation of the endoplasmic reticulum, low levels of ATP and the death of the parasite (Santi-Rocca et al., 2012). S-nitrosylation of *E. histolytica* cysteine proteinases, which are essential virulence factors, inactivate their activity (Siman-Tov and Ankri, 2003). S-nitrosylated (SNO) proteins may also be involved in the regulation of protein activity and function (Gould et al., 2013). Insights into the formation of SNO proteins and their regulation can be achieved by performing SNO-RAC analysis. This technique involves the capture of SNO proteins by chromatography on thiopropyl sepharose and mass spectrometry for their identification (Forrester et al., 2009). The heavy subunit of the *E. histolytica* Gal/GalNAc lectins was one of the SNO proteins identified by SNO RAC (Hertz et al., 2014a). S-nitrosylation occurred in the cysteine residues of the Gal/GalNAc lectin—carbohydrate recognition domain, which in turn disabled *E. histolytica* to attach to mammalian cells (Hertz et al., 2014a). The effects of NO on *E. histolytica* were also studied by transcriptomics. The most extensive groups of genes induced by NS are those related with signaling/regulatory processes, DNA repair, redox-regulation, glycolysis-related genes, and lipids (Vicente et al., 2009; Santi-Rocca et al., 2012). There is a significant overlap of genes modulated under NS and OS including genes involved in degradation and repair of misfolded proteins, lipid metabolism, transport and glycosylation, and DNA repair (Vicente et al., 2009). This overlap shows that the parasite uses common strategies to overcome the cytotoxicity of ROS and NOS (Vicente et al., 2009). Functional studies have demonstrated that Ehmeth is involved in the resistance of *E. histolytica* to NS (Hertz et al., 2014b). The authors proposed that enolase, a glycolytic protein that inhibits Ehmeth activity (Tovy et al., 2010), cannot bind to SNO-Ehmeth, thereby leading to higher tRNA^Asp^ methylation. NO resistance can also be achieved by the methylation of tRNA^Asp^ (Hertz et al., 2014b) and this requires more studies to elucidate the mechanism.

Infection with *E. histolytica* leads to non-symptomatic amoebiasis in 90% of the cases (Nath et al., 2015; Ishikane et al., 2016). Despite the absence of apparent inflammation, the parasite is nevertheless exposed to nanomolar levels of NO (Kolios et al., 2004). The exposure to low NO concentration can also occur due to molecules produced during the mechanism of denitrification by the microbiome present in the gut (Vermeiren et al., 2009). This non-toxic concentration of NO may prepare the parasite to resist higher concentration of NO. A recent study (Shahi et al., 2016b) has tested this hypothesis by adapting *E. histolytica* to a progressive amount of the NO donor S-nitrosoglutathione (GSNO) up to 120 μM. Here, the authors identified and studied the role of N-acetyl-ornithine deacetylase (NAOD) in aiding the parasite to adapt to NS. NAOD catalyzes the deacetylation of N-acetyl-L-ornithine to acetate and ornithine respectively. However, they found that the catalytic activity of this enzyme is not necessary to confer this protective effect on the parasite. Rather, NAOD has an interacting partner; glyceraldehyde 3-phosphate dehydrogenase (GAPDH). GAPDH has many roles apart from its role in metabolism (Nicholls et al., 2012). Its expression is increased in trophozoites exposed to NS and a high expression of this enzyme is detrimental to the parasite (Shahi et al., 2016b). Consequently, NAOD serves as a moonlighting protein that neutralizes the detrimental effect of GAPDH and helps the parasite to adapt to NS. Exciting pieces of evidence about the adaptation of the parasite to NS stem from SNO-RAC analysis (Trebicz-Geffen et al., 2017). Out of the SNO proteins in trophozoites adapted to NS, a significant enrichment of actin family cytoskeleton proteins was found. Trophozoites adapted to NO have their ability to form actin filaments impaired and consequently have their virulence reduced. These phenotypes are reversed upon removal of GSNO from the medium which suggests that the parasite has to compromise on some level for its adaptation to NS.

While *E. histolytica* depends on the NAOD-GAPDH interaction to neutralize the toxic effects NO, flavohemoglobin (flavoHb) in *Giardia intestinalis* reported to aerobically metabolize NO efficiently (Rafferty and Dayer, 2015). During NS, the expression of this protein is increased, and hence it aids in the breakdown of NO, whereas under normal conditions its expression is low.

## Global changes in the parasite in the absence of L-cysteine, iron, and glucose

### L-cysteine starvation

The parasite requires L-cysteine for its growth and survival. It obtains L-cysteine either through the extracellular medium or produces it via the de novo synthesis pathway with the help of Serine acetyl transferase (SAT) and cysteine synthase (CS) (Pye et al., 2004; Hussain et al., 2009). It is well known that cysteine takes part into the post-translational modifications of several proteins and its role also extends to redox mechanisms, electron transfer reactions, and many more processes (Beinert et al., 1997). The unique feature of this amino acid lies in the ability of its thiol group to endure redox reactions owing to its antioxidant property (Krauth-Siegel and Leroux, 2012). This feature makes cysteine extremely beneficial for the parasite which is deficient of antioxidant enzymes such as catalase and glutathione S-transferases. Cysteine is also essential for its growth, its adherence, and its resistance to OS (Jeelani et al., 2014) and also protects the parasite from the oxidative stress induced by the anti-amebic drug metronidazole (Leitsch et al., 2007). During the course of NS, the requirement of L-cysteine is increased, and the *de novo* pathway tries to compensate for increasing the production of cysteine. Yet, the amount of cysteine remains low in the cell and requires the addition of L-cysteine in the medium (Jeelani and Nozaki, 2014). Transcriptome analysis performed in the absence of L-cysteine revealed the upregulation of several genes belonging to the Fe-S cluster family of proteins. Among them, three iron-sulfur flavoproteins (EHI_025710, EHI_067720, and EHI_138480) showed higher expression in the absence of L-cysteine. Downregulation of EHI_025710 expression severely affected the growth of the parasite, whereas the downregulation of EHI_138480 showed a mild defect in the growth of *E. histolytica* in the absence of cysteine (Husain et al., 2011).

The deprivation of cysteine has also led to an increase in the intracellular concentration of metabolites such as O-acetyl serine sulfhydralase, glycerol-3-phosphate, and isopropanolamine (Table 1). Moreover, S-methyl cytosine levels were also increased in the cysteine-starved trophozoites, whereas under normal growth condition, S-methyl cytosine was undetectable (Husain et al., 2010). Cysteine deprivation has also caused an accumulation of phosphatidylisopropanolamine (Husain et al., 2011). The role of this unusual phospholipid is still unknown.

### Iron starvation

Iron is essential for the growth of *E. histolytica* (Park et al., 2001). Ferric ammonium citrate is provided to the parasite as the source of iron in the medium under laboratory conditions. In the host, the trophozoites scavenge iron from the bacterial flora and the iron-binding proteins of the host such as hemoglobin and ferratin by either phagocytosis or through hemophores or siderophores (Wandersman and Delepelaire, 2004). These proteins are released once the trophozoites phagocytose the cells (Lopez-Soto et al., 2009). Not much is known about the uptake, storage, and utilization of iron in *E. histolytica*. However, only a few proteins such as Rubrerythrin, NifS, and NifU are known to be involved in the iron-sulfur cluster formation (Ali et al., 2004; Maralikova et al., 2010). Iron is essential for the activity of proteins such as alcohol dehydrogenase 2, superoxide dismutase and ferredoxin (Tannich et al., 1991). Exposure to low iron concentrations *in vitro* impairs the parasite's adherence and cytopathic activity (Lee et al., 2008; Espinosa et al., 2009) suggesting the central role of iron toward the pathogenicity of the parasite. Iron may also affect the attachment of the parasite to the host cells along with cytotoxicity. Interestingly, there is a definite correlation between adherence, cytopathic activity and increasing concentration of iron. This effect is solely specific to iron and replacing the iron with other cationic salts did not reproduce this result (Lee et al., 2008). Transcriptome analysis of trophozoites growing in the absence of iron shows an increase in the transcription level of cysteine proteinases (CP-A5, CP-A7, and CP-EHI_01850), ribosomal proteins and elongation factors which are required for translation (Table 1). Iron deprivation also upregulates the expression of acyl-CoA synthetase, Androgen-Inducible Gene 1 (AIG1), ComEC protein, NADPH-dependent oxidoreductase (EhNO_2_) (Hernandez-Cuevas et al., 2014). The authors have also reported the higher expression of three genes belonging to the AIG1 family (EHI_022500, EHI_115160, and EHI_195260). A comparative study between two *Entamoeba histolytica* cell lines showed that these AIG1 genes were highly expressed in one of the cell lines that caused liver abscess in the gerbil model compared to the other which did not produce abscess (Biller et al., 2010). This observation suggests that AIG1 has a role in the virulence of the parasite. EhNO_2_ is involved in reducing cystine to cysteine as cysteine is normally present in the oxidized state inside the cells. Thus, its reduction is necessary before its utilization for various functions. EhNO_2_ also takes part in the reduction of metronidazole to activate this drug to produce reactive species that are toxic to the parasite (Jeelani et al., 2010). Three families of transporters were also upregulated in the absence of iron. ABC-family of transport proteins, P-glycoprotein-5 and Major family transporters (Hernandez-Cuevas et al., 2014). A similar family of transporters present in *Leishmania* is known for its ability to scavenge heme/iron from the medium (Perez-Victoria et al., 2001). An increase in the expression of these transporters in *E. histolytica* suggests that the parasite tries to acquire iron/heme from different sources during iron starvation. However, the role of these transporters in *E. histolytica* needs to be determined.

### Glucose starvation

*E. histolytica* resides the human colon, a niche where the amount of available glucose for fermentation is low (around 0.2 gr glucose/kg-1 tissue) due to the high absorptive capacity of the glucose transporters in the small intestine (Cummings and Macfarlane, 1997; Kellett et al., 2008; Hirayama et al., 2009). As a result, the parasite is facing glucose starvation (GS) in the human gut. The phenotypic and metabolic responses of *E. histolytica* to GS have been recently reviewed (Baumel-Alterzon and Ankri, 2014). When exposed to GS, the parasite downregulates the expression of genes involved in glycolysis and upregulates the genes involved in the degradation of stored products such as glycogen granules to make free glucose (Table 1). The research of new sources of energy by the parasite is illustrated by the upregulation of dihydropyrimidine dehydrogenase (DPD) expression. DPD is involved in the degradation of pyrimidines, and it is essential for the adaptation of *E. histolytica* to GS (Baumel-Alterzon et al., 2013). Although the exact role of DPD in the adaptation of *E. histolytica* to GS is still not understood, it is possible that the induction of DPD expression during glucose starvation contributes to energy production through the degradation of pyrimidines (Girjes et al., 1995; Beaulande et al., 1998). Metabolomics approaches that have been recently adapted to the study of *E. histolytica* metabolism (Jeelani and Nozaki, 2014) will help establish the existence of these pyrimidine degradation pathways in *E. histolytica*. Another example that illustrates the ability of the parasite to seek for an alternative source of carbon is the β-amylase-mediated degradation of mucin present in the colon (Thibeaux et al., 2013).

Another behavior associated with the exposure of the parasite to GS is its enhanced virulence. This phenotype correlates with the upregulation of some virulence factors like the Gal/GalNAc lectins and EhCP-A4, which is a cysteine protease that aids the parasite to invade the host by destroying the intracellular matrix (Baumel-Alterzon et al., 2013).

GS is also involved in epigenetic regulation by promoting the shuttling of the glycolytic enzyme enolase in the nucleus and the inhibition of Ehmeth (Tovy et al., 2011). The consequence of Ehmeth inhibition on the adaption of the parasite to GS is not yet understood.

## Human intestinal gut flora

The gut hosts a plethora of microbes, and their number exceeds 10^14^ (Thursby and Juge, 2017). It is estimated that about 400–1,000 bacterial species are present in the intestine, and 97% of the total population is made up by 30–40 species of bacteria (Xu et al., 2003; Sonnenburg et al., 2004; Jandhyala et al., 2015). The heterogeneous population of microbes inside the human gastrointestinal (GI) tract gives them the potential to influence human physiology in terms of promoting health and causing a wide variety of diseases such as obesity, irritable bowel syndrome and diarrhea, cardiovascular disorders, liver cirrhosis, to name a few (Chang and Lin, 2016). Upon entry into the host, *E. histolytica* finds itself in contact with a plethora of bacterial species. The intestinal lumen is the site of proliferation of the *E. histolytica* trophozoites, and they phagocytose the resident bacteria. Bacteria possessing specific recognition motifs were able to adhere to the parasite and to undergo ingestion (Bracha and Mirelman, 1984). The intricate relationship that exists between *E. histolytica* and the gut flora was the subject of many studies which concluded that it affects greatly several aspects of *E. histolytica* physiology. An axenic culture of *E. histolytica* replenished with *E. coli* O:55, was found to be more virulent or less virulent compared to the axenic culture alone depending on the time of interaction between the parasite and *E. coli* O:55 (Bracha et al., 1982; Mirelman, 1987; Padilla-Vaca et al., 1999). Moreover, it was found that bacteria exert their effect on *E. histolytica* virulence through cell surface entities such as lectin that attach to specific carbohydrate domains. One such gene coding for a 35-kDa lectin subunit of a heterodimeric Gal/GalNAc lectin molecule was found to be involved in promoting pathogenicity of the parasite, whereas the heavy chain subunit of 170 kDa was involved in adherence and attachment to the bacteria (Bhattacharya et al., 1992, 1998; Ankri et al., 1998; Padilla-Vaca et al., 1999). Apart from virulence, incubation with intestinal bacterial flora was suggested to trigger encystations in this parasite, which may explain why *E. histolytica* axenic cultures are unable to encyst *in vitro* (Ehrenkaufer et al., 2007). *E. histolytica* also has a direct influence on the gut microbiota composition (Verma et al., 2012). The presence of enteropathogenic bacteria (Paniagua et al., 2007) or the presence of *Prevotella copri* (Gilchrist et al., 2016), a normal component of the gut microbiota, has been correlated with *E. histolytica* infection. In contrast, the presence of *Clostridia* segmented filamentous bacteria is harmful to the parasite (Burgess et al., 2014).

## Concluding remarks

Appropriate adaptation to stress is essential for *E. histolytica*'s survival in harsh environments. In this review, we have shown that the parasite has developed multiple mechanisms regulated at the transcriptomics or at the post-transcriptomics level that allow an adequate response to a specific stress. While some features of the response of *E. histolytica* to stress are stress specific, some features such as the extensive reorganization of gene expression and expression of Hsps or antioxidant proteins such as thioredoxin are shared among different stresses. For years, studies about *E. histolytica*'s response to stresses have almost exclusively focused on the instant response of the parasite to acute stresses, but its ability to adapt to these stresses has not been sufficiently considered. Recent reports are illustrating the exceptional ability of this parasite to adapt to various stresses like GS, NS, and the role of DPD and NAOD proteins in the mechanism of adaptation (Baumel-Alterzon et al., 2013; Shahi et al., 2016b). We believe that strategies that counteract the protective effect of these proteins may be valuable in the struggle against this parasite. The identification of new targets for anti-amebic therapeutics will also beneficiate from a better integration of omics data available in the literature and of the ones to come. Multi-omic processing and combination of these data is a general challenge (Palsson and Zengler, 2010) that can only be overcome by training competent people to perform this task. Finally, it is essential not to neglect the procedures downstream to omics analysis of *E. histolytica* by improving current animal models of amoebiasis (Tsutsumi and Shibayama, 2006). This will enable us to reproduce the clinical manifestations observed in the human host in a better way. Moreover, the tools to knock out/downregulate gene expression can be enhanced (Morgado et al., 2016) by adapting CRISPR/Cas9 system to *E. histolytica* (Cui and Yu, 2016).

## Author contributions

All authors listed, have made substantial, direct and intellectual contribution to the work, and approved it for publication.

### Conflict of interest statement

The authors declare that the research was conducted in the absence of any commercial or financial relationships that could be construed as a potential conflict of interest. The handling editor is currently editing co-organizing a Research Topic with one of the author SA, and confirms the absence of any other collaboration.
